# Drawing a line: Differentiating mild from moderate dementia using the functional activities questionnaire^☆^^☆☆^

**DOI:** 10.1016/j.tjpad.2026.100630

**Published:** 2026-06-29

**Authors:** Ersin Ersözlü, Lukas Preis, Aykut Aktuz, Louise Droste, Akin Erman, Daria Gref, Katharina Sophie Strentz, Julian Hellmann-Regen, For the Alzheimer’s Disease Neuroimaging Initiative and the Frontotemporal Lobar Degeneration Neuroimaging Initiative

**Affiliations:** a Charité – Universitätsmedizin Berlin, Corporate Member of Freie Universität Berlin and Humboldt Universität zu Berlin, Department of Psychiatry and Neurosciences, Hindenburgdamm 30, 12203, Berlin, Germany; b Charité – Universitätsmedizin Berlin, Corporate Member of Freie Universität Berlin and Humboldt Universität zu Berlin, ECRC Experimental and Clinical Research Center, Lindenberger Weg 80, 13125, Berlin, Germany; c German Center for Neurodegenerative Diseases (DZNE) within the Helmholtz Association, Berlin, Germany; d Maastricht University, Faculty of Psychology and Neuroscience, Maastricht, the Netherlands

**Keywords:** Functional activities questionnaire, Dementia staging, Instrumental activities of daily living, Alzheimer's disease

## Abstract

**Background:**

Accurate differentiation between mild and moderate dementia is increasingly important, particularly as amyloid-targeting therapies are restricted to early disease stages. Functional impairment in instrumental activities of daily living is a hallmark of progression beyond mild dementia. The informant-based Functional Activities Questionnaire (FAQ) is widely used, but empirically validated cut-offs distinguishing mild from moderate dementia remain insufficiently defined.

**Methods:**

The optimal cut-off score was derived from the entire National Alzheimer’s Coordinating Center (NACC) Uniform Data Set as a discovery cohort (n = 34,513) and validated in two independent multicentric cohorts (Alzheimer’s Disease Neuroimaging Initiative (ADNI) n = 381 and Frontotemporal Lobar Degeneration Neuroimaging Initiative (FTLDNI) n = 74). The dementia staging was based on the Clinical Dementia Rating (CDR) global score. Functional impairment was assessed using the 10-item FAQ. Receiver operating characteristic analyses in NACC identified optimal thresholds, which were applied unchanged in validation cohorts. Sensitivity, specificity, positive predictive value, and negative predictive value were calculated, and discordant cases were examined to identify factors associated with misclassification.

**Results:**

In NACC, the FAQ demonstrated excellent discrimination of moderate dementia (AUC=0.947, 95% CI 0.944–0.949). A cut-off value of ≥18 maximized discrimination (sensitivity 96%, specificity 87%). A higher threshold of ≥23 improved specificity (92%), while maintaining sensitivity (83%). In ADNI and FTLDNI, the sensitivity of ≥18 threshold yielded 92% and 94%, respectively. Moreover, older age and lower cognitive performance were associated with higher odds of misclassification.

**Conclusions:**

The FAQ robustly differentiates mild from moderate dementia across diverse cohorts. A threshold of ≥18 prioritizes sensitivity, whereas ≥23 favors specificity, supporting context-dependent functional staging in clinical and research settings. Individuals with FAQ scores between 18 and 22 may benefit from more detailed clinical staging.

## Highlights

• The FAQ distinguishes mild from moderate dementia with high sensitivity.
• Two clinically interpretable cut-offs (≥18 and ≥23) are proposed.
• Thresholds were validated across independent multicenter cohorts.

## Introduction

The clinical introduction of disease-modifying anti-amyloid monoclonal antibodies has shifted the focus of dementia assessment toward early and precisely defined disease stages [1,2]. Current treatment indications are restricted to individuals with mild cognitive impairment (MCI) or mild dementia, emphasizing the need for reliable and easily applicable tools to distinguish mild from moderate dementia in routine clinical practice. Also, concomitant pathologies can impact clinical severity and heterogeneity [3,4], in terms of Alzheimer’s disease (AD) and related dementias, encompassing diverse etiologies (e.g., AD, frontotemporal lobar dementia [FTLD], Lewy body dementia, vascular dementia), each with distinct cognitive deficits and patterns of functional impairment [5].

Distinguishing mild from moderate dementia represents a critical but methodologically challenging task, while this transition corresponds to a clinically meaningful shift from partial independence to sustained functional dependence, with important implications for prognosis, caregiver burden, healthcare utilization, and eligibility for disease-modifying therapies and clinical trials. Despite its importance, operational definitions of this boundary remain heterogeneous, and commonly used cognitive measures often lack precision in distinguishing clinical stages.

Cognitive screening instruments such as the Mini-Mental State Examination (MMSE) [6] are widely used to characterize dementia severity, while global staging tools to assess both cognition and functioning, such as the Clinical Dementia Rating (CDR), require longer administration time and may therefore be less practical in a routine clinical setting [[7], [8], [9], [10]]. However, cognition-based staging is vulnerable to ceiling and floor effects and non-linear patterns of decline.

Functional impairment in instrumental activities of daily living (IADLs) represents both an early predictor and a key marker of disease progression beyond the mild dementia stage [[10], [11], [12], [13]]. Informant-based assessments of everyday function, therefore, play a central role in dementia diagnosis and staging [[14], [15], [16]]. The Functional Activities Questionnaire (FAQ) is widely used in both research and clinical settings and provides a pragmatic assessment of IADLs, including financial management, medication adherence, shopping, and transportation [17,18]. The FAQ scores increase consistently across dementia severity levels and exhibit excellent discriminatory accuracy across multiple contrasts, supporting the use of the FAQ total score as a reliable and scalable measure of functional severity, with strong internal consistency, test–retest reliability, and generalizability [15,18,19].

Although established cut-offs exist for identifying functional impairment [14], the specific utility of the FAQ for differentiating mild versus moderate dementia remains insufficiently defined. We expect that the FAQ, as an informant-based approach, provides a robust functional assessment also among dementia stages [15,20]. The present study, therefore, aims to derive and validate clinically meaningful FAQ cut-offs for distinguishing mild from moderate dementia using a discovery–validation framework across independent multicenter cohorts.

## Methods

### Study design

This study used a two-stage discovery–validation design. The optimal FAQ cut-off value for distinguishing mild from moderate-to-severe dementia was derived in the discovery cohort (i.e., National Alzheimer’s Coordinating Center [NACC]) and evaluated in two independent validation cohorts (i.e., AD Neuroimaging Initiative [ADNI] and the Frontotemporal Lobar Degeneration Neuroimaging Initiative [FTLDNI]).

### Study data sources

Data were obtained from the NACC Uniform Data Set (UDS), which includes standardized clinical evaluations, neuropsychological testing, informant-based functional assessments, and neuropathological data collected across U.S. Alzheimer’s Disease Research Centers. All contributing centers obtained local institutional review board approval, and all participants or their representatives provided informed consent.

Data used in the preparation of this article were obtained from the ADNI database (adni.loni.usc.edu). The ADNI was launched in 2003 as a public-private partnership, led by Principal Investigator Michael W. Weiner, MD. The original goal of ADNI was to test whether serial magnetic resonance imaging, positron emission tomography, other biological markers, and clinical and neuropsychological assessment can be combined to measure the progression of MCI and early AD. The current goals include validating biomarkers for clinical trials, improving the generalizability of ADNI data by increasing diversity in the participant cohort, and to provide data concerning the diagnosis and progression of AD to the scientific community. For up-to-date information, see adni.loni.usc.edu.

FTLDNI was funded through the National Institute of Aging, and started in 2010. The primary goals of FTLDNI were to identify neuroimaging modalities and methods of analysis for tracking FTLD and to assess the value of imaging versus other biomarkers in diagnostic roles. The Principal Investigator of NIFD was Dr. Howard Rosen, MD at the University of California, San Francisco. The data are the result of collaborative efforts at three sites in North America. For up-to-date information on participation and protocol, please visit http://memory.ucsf.edu/research/studies/nifd.

### Study participants

Participants with available data for the FAQ and CDR global score were included in the study. For both ADNI and FTLDNI, a single visit per participant was analyzed, defined as the first visit that met criteria for moderate dementia when applicable, or, otherwise, the earliest visit assessment. The final cohorts included n = 34,513 for NACC UDS (entire available baseline dataset without restriction for clinical stages), n = 381 for ADNI (n = 143 for mild dementia and n = 238 for moderate dementia), and n = 74 for FTLDNI (n = 24 for mild dementia, n = 49 for moderate dementia, and n = 1 for severe dementia). Importantly, the discovery cohort included participants across the cognitive spectrum to ensure robust estimation of discrimination thresholds.

### Clinical diagnosis, staging and cognitive screening

Clinical etiologic diagnoses were cohort-specific and reflected the primary design goals of each study. The NACC UDS captures real-world, multi-etiology clinical diagnoses assigned at Alzheimer’s Disease Centers using contemporaneous consensus criteria, allowing multiple contributing etiologies (e.g., AD with vascular or Lewy body contributions) and variable biomarker availability across sites and calendar time. Participants are defined as cognitively impaired when they are diagnosed with dementia, MCI (either amnestic or non-amnestic), or other impairment (cognitively impaired but who do not meet the criteria for MCI). In contrast, the ADNI applies protocol-defined clinical inclusion criteria focused on the AD continuum. FTLDNI is etiology-focused on frontotemporal lobar degeneration syndromes, enrolling participants meeting syndrome-specific consensus criteria (behavioral-variant [bv-FTLD] and primary progressive aphasia [PPA] variants) or diagnosed as not otherwise specified FTLD. Because baseline visits span different calendar periods and waves (NACC UDS Form Versions 1–3 and ADNI-1, ADNI-GO, ADNI-2, ADNI-3) and criteria and biomarker practices evolved over time, analyses accounted for cohort- and wave-related differences in diagnostic operationalization and severity distributions. We summarized the diagnostic criteria for etiologic diagnosis in **Supplementary Table S1**.

Importantly, dementia severity was harmonized using the CDR global score to minimize circularity with functional outcome measures [10]. Mild dementia was defined as a CDR global score of 1, and moderate dementia as a CDR global score of 2.

The MMSE was included as a cognitive severity measure in baseline characteristics [6]. In the NACC cohort, as the discovery sample, we conducted sensitivity analyses to further examine concordance with MMSE ranges (MMSE≥21 for mild dementia; MMSE 10–20 for moderate dementia, and MMSE<10 for severe dementia) [21], resulting in an alternative definition of moderate dementia based on CDR>1 and MMSE<21. Importantly, validation of cohort analyses relied solely on CDR global scores, as in the discovery analysis.

### Functional assessment

Functional status was assessed using the FAQ, a 10-item informant-based instrument evaluating IADLs [17]. Each item is scored from 0 to 3, yielding a total score range of 0–30, with higher scores indicating greater functional impairment. The FAQ total scores were analyzed as continuous measures, with thresholds used for classification.

### Statistical analysis

All analyses were conducted using SPSS version 31 (IBM Corp., Armonk, NY). Baseline demographic and clinical characteristics were summarized and stratified by study cohort and clinical stage. Continuous variables were reported as means ± standard deviations and categorical/ordinal variables as counts and percentages. The Kruskal-Wallis test or the Mann-Whitney U test for continuous variables and the chi-squared test for categorical variables were used for group comparisons. Between-cohort differences in baseline characteristics were quantified using standardized mean differences (SMDs), calculated using pooled standard deviations for continuous variables and Cohen’s h for categorical variables [22]. All tests were two-sided with a significance threshold of p < 0.05.

Receiver operating characteristic (ROC) analyses evaluated the ability of FAQ scores to discriminate between mild and moderate dementia. Discriminative performance was quantified using the area under the ROC curve (AUC). The optimal FAQ cut-off in the discovery cohort was identified using Youden’s index. Prespecified FAQ cut-offs derived in the discovery cohort were applied without modification in ADNI and FTLDNI. Positive predictive value (PPV), negative predictive value (NPV), sensitivity, and specificity were reported.

To examine factors associated with discordance between FAQ- and CDR-based staging, we conducted separate logistic regression analyses within CDR-defined severity strata. Among participants with CDR<2, predictors of false-positive (FP) classification (FAQ ≥ primary cut-off despite mild dementia) were evaluated relative to true negatives. Among participants with CDR≥2, predictors of false-negative (FN) classification (FAQ < primary cut-off despite moderate dementia) were evaluated relative to true positives. Separate multivariable logistic regression models were fitted for FN and FP classification. Age, sex (female vs. male), MMSE score, cognitive impairment status (cognitively impaired vs. cognitively normal), self-reported race (White vs. other racial groups) and ethnicity (Hispanic vs. non-Hispanic), as well as living (alone vs. not alone) and marital status (not married vs. married) were included in both models as independent variables. Further multivariable logistic regression analyses were conducted to examine whether the total FAQ score remained independently associated with moderate dementia after adjustment for these socio-demographic confounders. In additional analyses restricted to participants with cognitive impairment, probable AD was included as an additional independent variable.

To examine the relationship between functional impairment and clinical severity, cross-tabulation analyses were used to assess the association between categorized FAQ levels (independent variable) and both CDR stages and MMSE categories as dependent variables, with separate ordinal asymmetric associations evaluated using linear-by-linear χ² tests and Somers’ D statistics. Sensitivity analyses in the discovery cohort assessed robustness of discrimination performance at the primary FAQ cut-off across alternative staging definitions (CDR-only vs. CDR combined with MMSE thresholds) and after separate restriction to participants with a clinical diagnosis of probable AD and to those with cognitive impairment without AD (non-AD). Further sensitivity analyses restricted the non-AD subgroup to participants with dementia severity of at least CDR global =1 to examine whether including earlier-stage impairment influenced subgroup discrimination characteristics.

## Results

### Cohort characteristics

Baseline demographic and clinical characteristics are summarized in Table 1 and detailed in **Supplementary Table S2**, stratified by cohort and dementia severity. Age demonstrated a moderate to large imbalance, with the highest mean age in ADNI, followed by NACC, and the lowest in FTLDNI. Educational attainment showed a small imbalance only between ADNI and FTLDNI. Sex distribution showed a small to moderate imbalance, with a higher proportion of female participants in NACC than in ADNI or FTLDNI. Participants in NACC revealed a more diverse racial and ethnic composition. The proportion of participants with moderate/severe dementia showed a very large imbalance between NACC and both external cohorts, whereas ADNI and FTLDNI revealed minimal imbalance. Within cohorts, participants with moderate/severe dementia were older than those without in both NACC (73.4 vs 70.6 years, p < 0.001) and FTLDNI (65.4 vs 60.8 years, p = 0.006). Education was slightly lower in NACC among those with moderate/severe dementia (p < 0.001). Within the NACC cohort, sex, ethnicity, and race were weakly associated with moderate dementia status (all p < 0.001), while ADNI and FTLDNI revealed no demographical differences across severity groups (**Supplementary Table S2**).

**Table 1:** Baseline characteristics of participants by cohort. Values are presented as mean ± SD for continuous variables and percentages for categorical variables.

**Characteristic**	**NACC (N=34,513)**	**ADNI (N=381)**	**FTLDNI (N=74)**	**p**	**SMD/Cohen’s h NACC–ADNI**	**SMD/Cohen’s h NACC–FTLDNI**	**SMD/Cohen’s h ADNI–FTLDNI**
Age, years	70.8 ± 10.5	74.8 ± 7.7	63.9 ± 6.5	<0.001	0.39	0.69	1.47
Education, years	15.4 ± 3.2	15.4 ± 2.8	15.8 ± 3	0.64	0	0.13	0.14
Female, %	61.2	45.4	39.2	<0.001	0.32	0.46	0.13
White, %	79.4	93.2	91.9	<0.001	0.36	0.31	0.04
Black/African American, %	14.9	4.2	0	<0.001	0.36	0.82	0.46
Asian, %	2.9	1.6	6.8	<0.001	0.09	0.30	0.25
Cognitive impairment, %	54	100	100	n.a.	n.a.	n.a.	n.a.
Clinical etiologic diagnosis of AD, %	32.7	100	0	n.a.	n.a.	n.a.	n.a.
CDR global score of ≥2, %	9	62.5	67.6	<0.001	1.20	1.33	0.12

Abbreviations: n.a., not applicable; SD, standard deviation; SMD, standardized mean difference.

### The identification of cut-off and discrimination of moderate dementia in the discovery cohort

**Fig. 1:**
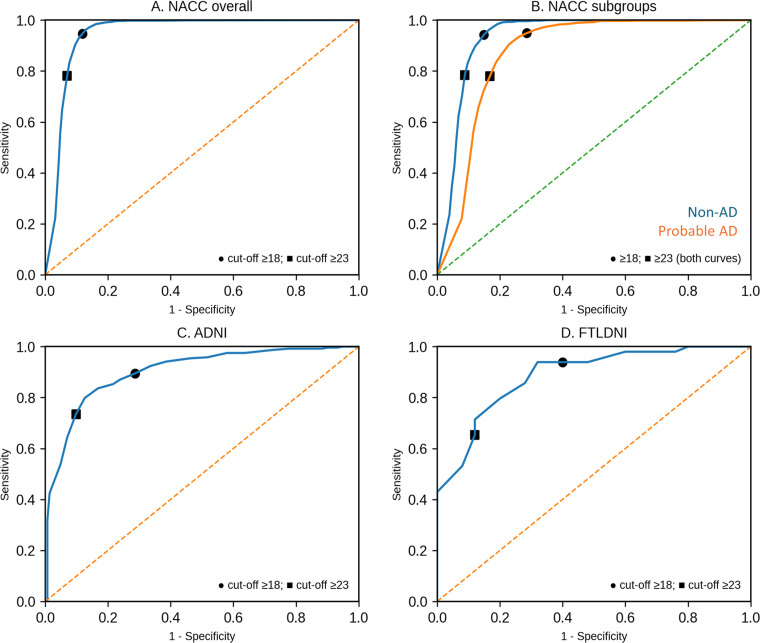
ROC curves of the FAQ total score for discrimination of moderate dementia (CDR ≥2) across discovery and validation cohorts. (A) NACC overall; (B) NACC probable AD and non-AD; (C) ADNI; (D) FTLDNI. Circles denote the prespecified cut-off ≥18, and squares denote ≥23.

In the NACC cohort (n = 34,513), which included participants across pre-dementia and dementia stages, the FAQ demonstrated excellent discrimination of moderate dementia at baseline (area under the curve [AUC] = 0.947, 95% CI 0.944–0.949) (Fig. 1A). A cut-off of ≥18 yielded the highest Youden’s index (0.83), corresponding to 96% sensitivity and 87% specificity (Table 2). At this threshold, the negative predictive value (NPV) was 99.6%, and the positive predictive value (PPV) was 42.2% (**Supplementary Table S3, Supplementary Figure 1A**).

**Table 2:** Diagnostic performance of the FAQ total score across all cut-off scores for the identification of moderate dementia in the NACC Cohort, including overall cohort and probable AD and non-AD subgroups.

	**NACC (overall)**			**NACC (probable AD)**			**NACC (non-AD)**		
**Cut-off**	**Sensitivity (%)**	**Specificity (%)**	**Youden’s Index**	**Sensitivity (%)**	**Specificity (%)**	**Youden’s Index**	**Sensitivity (%)**	**Specificity (%)**	**Youden’s Index**
≥1	0.999	0.556	0.555	0.999	0.157	0.156	1	0.358	0.358
≥2	0.998	0.613	0.611	0.998	0.209	0.207	1	0.438	0.438
≥3	0.998	0.653	0.651	0.998	0.254	0.252	1	0.509	0.509
≥4	0.998	0.679	0.677	0.998	0.291	0.289	1	0.556	0.556
≥5	0.998	0.701	0.699	0.998	0.327	0.324	1	0.594	0.594
≥6	0.998	0.719	0.717	0.998	0.359	0.357	1	0.622	0.622
≥7	0.998	0.736	0.734	0.997	0.391	0.389	1	0.649	0.649
≥8	0.997	0.751	0.748	0.997	0.421	0.418	0.998	0.671	0.669
≥9	0.997	0.765	0.762	0.997	0.45	0.447	0.998	0.692	0.69
≥10	0.996	0.777	0.773	0.997	0.477	0.474	0.996	0.71	0.705
≥11	0.993	0.789	0.782	0.992	0.503	0.495	0.996	0.727	0.723
≥12	0.992	0.801	0.793	0.991	0.529	0.52	0.996	0.742	0.738
≥13	0.99	0.813	0.803	0.989	0.556	0.545	0.993	0.762	0.756
≥14	0.987	0.826	0.813	0.985	0.583	0.569	0.993	0.779	0.773
≥15	0.985	0.837	0.822	0.983	0.61	0.594	0.991	0.793	0.784
≥16	0.979	0.847	0.827	0.978	0.634	0.612	0.985	0.807	0.791
≥17	0.972	0.859	0.831	0.972	0.661	0.632	0.974	0.821	0.795
≥18	0.963	0.87	0.832	0.963	0.686	0.649	0.961	0.836	0.797
≥19	0.947	0.881	0.829	0.949	0.714	0.663	0.943	0.851	0.794
≥20	0.931	0.892	0.823	0.934	0.74	0.674	0.921	0.863	0.784
≥21	0.904	0.904	0.808	0.906	0.77	0.676	0.897	0.879	0.777
≥22	0.867	0.914	0.781	0.867	0.794	0.661	0.865	0.892	0.756
≥23	0.832	0.923	0.754	0.833	0.813	0.645	0.83	0.903	0.732
≥24	0.781	0.93	0.711	0.78	0.832	0.612	0.784	0.912	0.696
≥25	0.717	0.938	0.655	0.722	0.852	0.574	0.701	0.921	0.622
≥26	0.648	0.946	0.593	0.655	0.869	0.524	0.624	0.932	0.556
≥27	0.558	0.952	0.51	0.567	0.884	0.451	0.528	0.939	0.467
≥28	0.44	0.957	0.397	0.444	0.897	0.341	0.428	0.945	0.373
≥29	0.34	0.962	0.303	0.338	0.909	0.247	0.349	0.953	0.303
≥30	0.223	0.968	0.192	0.219	0.922	0.142	0.236	0.961	0.197

Abbreviations: AD, Alzheimer’s disease.

In a subsample of participants with cognitive impairment and an etiologic diagnosis of AD (n = 11,300), the FAQ revealed good discrimination between moderate and less severe impairment (AUC = 0.873, 95% CI 0.866–0.880; Fig. 1B). At the predefined cut-off (≥18), sensitivity was 96%, and specificity was 69%, yielding a PPV of 44.6% and an NPV of 98.5% within this subgroup (**Supplementary Table S3**). In a subsample of cognitively impaired participants without an etiologic diagnosis of AD (n = 7358), the FAQ demonstrated an excellent discrimination between moderate and less severe impairment (AUC = 0.934, 95% CI 0.927–0.940; Fig. 1B). At the predefined cut-off (≥18), sensitivity was 96%, and specificity was 84%, corresponding to a PPV of 28.5% and an NPV of 99.7% within this subgroup (**Supplementary Table S3**). To address the possible impact of MCI in the non-AD group, we repeated the analyses after restricting the etiologic diagnostic groups to participants with CDR global ≥1, which comprised 51% of the probable AD group and 24.7% of the non-AD group. Here, ROC analyses achieved comparable discrimination across the AD and non-AD groups (AUC_AD_ = 0.724 ± 0.007 (95% CI 0.711–0.737) vs. AUC_non-AD_ = 0.695 ± 0.012 [95% CI 0.671–0.719]) (**Supplementary Table S4**).

### Discrimination in validation cohorts

In the external validation cohort from ADNI (n = 381), application of the predefined cut-off demonstrated strong discrimination between mild and moderate dementia (AUC = 0.902, 95% CI 0.870–0.933; Fig. 1C). Sensitivity was 92%, and specificity was 66% (Table 3), corresponding to a PPV of 82.1% and an NPV of 84.1% (**Supplementary Table S3**).

**Table 3:** Diagnostic performance of the FAQ total score across selected cut-off scores in the independent validation cohorts (ADNI and FTLDNI). The table includes only clinically relevant cut-off values to improve clarity and focus on meaningful discrimination thresholds.

	**ADNI**			**FTLDNI**		
**Cut-off**	**Sensitivity (%)**	**Specificity (%)**	**Youden’s Index**	**Sensitivity (%)**	**Specificity (%)**	**Youden’s Index**
≥11	0.992	0.224	0.215			
≥12	0.987	0.273	0.26	1	0.08	0.08
≥13	0.975	0.364	0.338	1	0.16	0.16
≥14	0.975	0.42	0.394	1	0.2	0.2
≥15	0.958	0.483	0.441	0.98	0.24	0.22
≥16	0.954	0.538	0.492	0.98	0.4	0.38
≥17	0.941	0.615	0.557			
≥18	0.924	0.664	0.589	0.939	0.52	0.459
≥19	0.895	0.713	0.608	0.939	0.6	0.539
≥20	0.87	0.762	0.632	0.939	0.68	0.619
≥21	0.853	0.783	0.636	0.857	0.72	0.577
≥22	0.836	0.832	0.668	0.796	0.8	0.596
≥23	0.798	0.874	0.672	0.714	0.88	0.594
≥24	0.735	0.902	0.637	0.653	0.88	0.533
≥25	0.643	0.93	0.573	0.531	0.92	0.451
≥26	0.538	0.951	0.489	0.429	1	0.429
≥27	0.424	0.986	0.41	0.367	1	0.367
≥28	0.319	0.993	0.312	0.184	1	0.184
≥29	0.185	0.993	0.178	0.082	1	0.082
≥30	0.097	0.993	0.09	0.041	1	0.041

In FTLDNI (n = 74), the FAQ achieved an AUC of 0.886 (95% CI 0.809–0.963; Fig. 1D). Sensitivity for identifying moderate-to-severe dementia was 94%, and specificity was 52%, resulting in a PPV of 79.6% and an NPV of 80.0% (**Supplementary Table S3**).

### An alternative specificity-prioritizing cut-off and transitional functional range

To increase specificity, an alternative threshold of ≥23 was examined (Table 2). In the NACC cohort, this cut-off yielded 83% sensitivity and 92% specificity, with a PPV of 51.0% and an NPV of 98.2% (**Supplementary Table S3**). In ADNI, the ≥23 threshold achieved 80% sensitivity and 87% specificity, corresponding to a PPV of 90.7% and an NPV of 73.6% (Table 3, **Supplementary Table S3**). In FTLDNI, sensitivity was 71% and specificity was 88%, yielding a PPV of 92.5% and an NPV of 57.0% (Table 3, **Supplementary Table S3**).

**Fig. 2:**
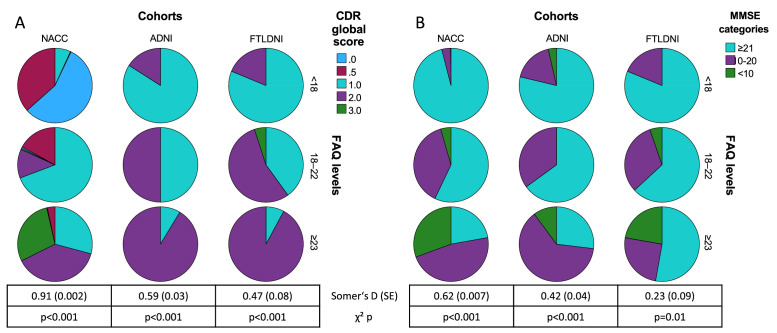
Distribution of clinical severity across categorized FAQ levels. (A) Distribution of CDR global stages across FAQ levels and (B) distribution of MMSE categories across FAQ levels among the NACC, ADNI, and FTLDNI cohorts.

Abbreviations: FAQ, Functional Activities Questionnaire; CDR, Clinical Dementia Rating; MMSE, Mini–Mental State Examination; SE, standard error.

To further characterize the functional continuum between mild and moderate dementia, FAQ scores were categorized into three ordinal levels (≤17, 18–22, and ≥23) and compared with CDR global stage and MMSE categories. Higher FAQ levels were consistently associated with greater clinical severity (Fig. 2). Cross-tabulation analyses demonstrated moderate-to-strong ordinal associations between FAQ levels and CDR global stage (Somers’ D = 0.91 [SE 0.002] in NACC; 0.59 [SE 0.03] in ADNI; and 0.47 [SE 0.08] in FTLDNI; all p < 0.001). A similar gradient was observed between FAQ levels and MMSE categories, with higher functional impairment corresponding to lower cognitive performance, although the strength of the association varied across cohorts (Somers’ D = 0.62 [SE 0.007] in NACC, 0.42 [SE 0.04] in ADNI, and 0.23 [SE 0.09] in FTLDNI; p < 0.001 for NACC and ADNI, p = 0.01 for FTLDNI).

### Predictors of discordant classification and adjusted prediction models

To examine predictors of discordance between FAQ- and CDR-based staging at the FAQ cut-off of ≥18, we conducted separate multivariable logistic regression analyses for FN and FP classifications in the NACC cohort (**Supplementary Table S5**). While FN classifications were uncommon, a lower MMSE score was the only consistent predictor of FN classification in both the overall cohort (Odds Ratio [OR]: 0.934 [95% CI 0.908–0.961], p < 0.001) and the cognitively impairment-restricted sample (OR: 0.940 [95% CI 0.912–0.968], p < 0.001). In contrast, FP classifications were more frequent and were associated with older age (OR: 1.012 [95% CI 1.007–1.017], p < 0.001), lower MMSE score (OR: 0.966 [95% CI 0.944–0.971], p < 0.001), and cognitive impairment (OR: 107.9 [95% CI 63.6–183.1], p < 0.001). Living status and marital status were also associated with discordant classification, as participants living alone (OR: 0.45 [95% CI 0.384–0.527], p < 0.001) and those who were married (OR: 0.783 [95% CI 0.693–0.884], p < 0.001) showed reduced odds of FP classification. In contrast, participants identified as White (OR: 1.386 [95% CI 1.216–1.580], p < 0.001) had increased odds of FP classification. In dementia-restricted analyses, probable AD diagnosis was additionally associated with higher odds of FP classification (OR: 1.911 [95% CI 1.711–2.136], p < 0.001).

In additional adjusted analyses, each one-point increase in FAQ score remained independently associated with higher odds of moderate dementia in both the overall NACC cohort (OR 1.465, 95% CI 1.444–1.487, p < 0.001; Nagelkerke R²=0.756) and the cognitively impaired subgroup (OR 1.464, 95% CI 1.442–1.486, p < 0.001; Nagelkerke R²=0.723), after adjustment for age, sex, race, ethnicity, marital status, and living status (**Supplementary Table S6**).

### Alternative reference staging

In sensitivity analyses using the alternative CDR & MMSE (combined) definition of moderate dementia, the FAQ showed similarly strong discrimination (AUC = 0.949, 95% CI 0.946–0.952), while the optimal threshold corresponded to FAQ ≥ 18 (sensitivity 97.2%, specificity 86.5%), consistent with the primary analyses, suggesting that the FAQ cut-off was not materially dependent on the specific operational definition of moderate dementia (**Supplementary Table S7**).

## Discussion

The present study evaluated the ability of the FAQ to differentiate between mild and moderate dementia across large, multicenter cohorts using a discovery–validation framework. Across cohorts, FAQ scores demonstrated strong discrimination between these stages, supporting the utility of informant-based functional assessment for staging dementia severity in both research and clinical contexts. The identified thresholds provide a practical framework for interpreting functional impairment and may facilitate more consistent differentiation between mild and moderate disease stages in AD and related dementias. This distinction is increasingly relevant in the context of contemporary therapeutic strategies for AD, where accurate clinical staging is essential for treatment eligibility and appropriate patient selection [2].

The findings were robust across sensitivity analyses applying alternative staging definitions and etiologic restrictions. Similar discrimination patterns were observed when analyses were split into probable AD and cognitive impairment of other causes, supporting the generalizability of the identified thresholds across clinical contexts. Notably, subgroup analyses indicated higher specificity within the broader non-AD group for moderate dementia at the FAQ cut-off of ≥18 in non-AD populations compared to probable AD. While no similar analyses are available to date, prior evidence suggests that functional impairment may vary across dementia etiologies. A previous study using the Disability Assessment for Dementia demonstrated that bv-FTLD is associated with greater functional impairment—particularly in basic activities of daily living (BADLs)—compared to AD and other FTLD subtypes, with limited correlation with cognitive measures or global staging scales such as the CDR [23]. Furthermore, another study examining the implementation of FAQ in the NACC cohort across FTLD subtypes similarly found that bv-FTLD differed from semantic and non-fluent PPA variants, while higher FAQ scores correlated with more severe behavioral symptoms and lower executive functioning across all subtypes [19]. Also, Lewy body dementia diagnosis has been linked to more advanced functional impairment in BADLs compared to AD [24].

Functional impairment in IADLs represents a central feature of dementia progression and complements cognitive measures when evaluating disease severity [15]. The FAQ provides an efficient method for capturing these functional changes and has demonstrated strong reliability and validity across large clinical datasets [14,18]. From a clinical implementation perspective, the FAQ offers a pragmatic, scalable approach to functional staging in time-constrained clinical environments where comprehensive staging instruments may be impractical. Rather than replacing established staging procedures, the FAQ may serve as an efficient screening assessment that can guide a more detailed evaluation. A threshold of ≥18 prioritizes high sensitivity and negative predictive value, supporting its utility as a screening-oriented functional staging approach. However, negative predictive values were highest in the NACC cohort and notably lower in the external validation cohorts, suggesting that rule-out performance may vary by cohort composition and dementia stage prevalence. Scores between 18 and 22 likely represent a transitional range of functional impairment and warrant comprehensive staging with instruments such as the CDR scale. Even at higher cut-off scores (≥23), which improve specificity and positive predictive value, clinical confirmation remains essential.

The ordinal analyses further supported the validity of the identified thresholds by demonstrating a clear monotonic relationship between functional impairment and both global dementia staging and cognitive performance. Higher FAQ levels were consistently associated with more advanced clinical severity, whereas the intermediate range between 18 and 22 showed a heterogeneous distribution across clinical stages. This pattern supports interpreting this interval as a transitional range of functional decline rather than a discrete stage of disease severity.

Discordance between FAQ- and CDR-based staging was asymmetrical, with FP classifications occurring more frequently than FN. FN cases were rare and consistently associated with lower MMSE scores, suggesting that advanced cognitive impairment is not always accompanied by proportionally greater informant-reported functional decline, potentially reflecting residual functional reserve or underreporting. In contrast, FP classifications were associated with older age and lower MMSE, but also with diagnostic and staging heterogeneity, including individuals with cognitively impairment and those with probable AD. These seemingly divergent associations likely reflect distinct mechanisms underlying elevated FAQ scores across subgroups rather than a single, coherent phenotype. While prior work suggests that functional impairment may exceed global cognitive staging, particularly in non-AD neurodegenerative disorders, our findings do not directly contradict this notion but rather highlight the influence of cohort composition [25]. Specifically, the inclusion of individuals with predementia stages within the non-AD group in the NACC cohort may attenuate the expected pattern of disproportionate functional decline. This possible interpretation was supported by sensitivity analyses restricting the non-AD subgroup to participants with CDR global ≥1, which revealed broadly comparable discrimination between the AD and non-AD subgroups. Additionally, aggregating heterogeneous IADL domains into the FAQ total score may further contribute to discordance, as different items capture distinct cognitive and non-cognitive aspects of functional impairment [25,26].

Several strengths of the present study should be noted. The large discovery cohort enabled stable estimation of discrimination metrics and thresholds across the spectrum of cognitive impairment. Independent validation in cohorts with distinct recruitment strategies and clinical phenotypes strengthens the generalizability of the findings. In addition, discordance analyses provided insight into circumstances in which functional and global staging measures may diverge.

Several limitations should be considered. First, functional assessments rely on informant reports and may be influenced by informant characteristics or contextual factors. Especially, FAQ, as an informant-based IADL measure, may be influenced by historical gender-role patterns and cohort effects, which could affect responses to specific items and potentially attenuate comparability across genders and generations. Second, because the FAQ primarily captures IADLs rather than BADLs, it may miss clinically relevant declines in BADLs. Third, the included cohorts were classified primarily using clinical diagnostic criteria, whereas current disease-modifying therapy eligibility generally requires biomarker confirmation of cerebral amyloid pathology and is limited to early symptomatic disease; therefore, our findings should not be interpreted as a direct treatment-allocation algorithm. Most participants were recruited in specialized research settings, which may limit generalizability to community populations. Racial and ethnic diversity across cohorts was limited. Due to small sample sizes for several racial groups, particularly in the validation cohorts, race was harmonized for analysis as White versus other racial groups. Furthermore, the cross-sectional design does not allow conclusions regarding longitudinal progression or transitions between dementia stages.

Future research should evaluate the longitudinal performance of the FAQ in capturing transitions between dementia stages and examine how functional trajectories relate to cognitive decline and biomarker-defined disease progression [27], the latter being especially relevant in the context of disease-modifying treatments [1]. Replication in more diverse populations and community-based settings will be important to strengthen generalizability, as sex, affective symptoms, and age-related factors such as comorbidity and sensory limitations may further confound functional assessments independent of neurodegeneration [[28], [29], [30], [31], [32], [33]], and their impact should be further examined.

## Funding

No funding was received for this study.

## Declaration of generative AI and AI-assisted technologies in the writing process

No artificial intelligence tools or AI-assisted technologies were used in the preparation of this manuscript.

## CRediT authorship contribution statement

**Ersin Ersözlü:** Writing – review & editing, Writing – original draft, Visualization, Validation, Formal analysis, Data curation, Conceptualization. **Lukas Preis:** Writing – review & editing, Conceptualization. **Aykut Aktuz:** Writing – review & editing. **Louise Droste:** Writing – review & editing. **Akin Erman:** Writing – review & editing. **Daria Gref:** Writing – review & editing. **Katharina Sophie Strentz:** Writing – review & editing. **Julian Hellmann-Regen:** Writing – review & editing, Supervision.

## Declaration of competing interest

The authors declare that there are no known competing financial interests or personal relationships that could have influenced the work reported in this manuscript.

## Appendix Supplementary materials

Image, application 1
